# Integrated examination of the transcriptome and metabolome of the gene expression response and metabolite accumulation in soybean seeds for seed storability under aging stress

**DOI:** 10.3389/fpls.2024.1437107

**Published:** 2024-07-08

**Authors:** Guang Li, Jianguo Xie, Wei Zhang, Fanfan Meng, Mingliang Yang, Xuhong Fan, Xingmiao Sun, Yuhong Zheng, Yunfeng Zhang, Mingliang Wang, Qingshan Chen, Shuming Wang, Hongwei Jiang

**Affiliations:** ^1^ Jilin Academy of Agricultural Sciences (China Agricultural Science and Technology Northeast Innovation Center), Soybean Research Institute, Changchun, China; ^2^ Northeast Agricultural University, Harbin, Heilongjiang, China

**Keywords:** soybean, aging, caffeic acid, transcriptome, metabolomic

## Abstract

Soybean quality and production are determined by seed viability. A seed’s capacity to sustain germination via dry storage is known as its seed life. Thus, one of the main objectives for breeders is to preserve genetic variety and gather germplasm resources. However, seed quality and germplasm preservation have become significant obstacles. In this study, four artificially simulated aging treatment groups were set for 0, 24, 72, and 120 hours. Following an aging stress treatment, the transcriptome and metabolome data were compared in two soybean lines with notable differences in seed vigor—R31 (aging sensitive) and R80 (aging tolerant). The results showed that 83 (38 upregulated and 45 downregulated), 30 (19 upregulated and 11 downregulated), 90 (52 upregulated and 38 downregulated), and 54 (25 upregulated and 29 downregulated) DEGs were differentially expressed, respectively. A total of 62 (29 upregulated and 33 downregulated), 94 (49 upregulated and 45 downregulated), 91 (53 upregulated and 38 downregulated), and 135 (111 upregulated and 24 downregulated) differential metabolites accumulated. Combining the results of transcriptome and metabolome investigations demonstrated that the difference between R31 and R80 responses to aging stress was caused by genes related to phenylpropanoid metabolism pathway, which is linked to the seed metabolite caffeic acid. According to this study’s preliminary findings, the aging-resistant line accumulated more caffeic acid than the aging-sensitive line, which improved its capacity to block lipoxygenase (LOX) activity. An enzyme activity inhibition test was used to demonstrate the effect of caffeic acid. After soaking seeds in 1 mM caffeic acid (a LOX inhibitor) for 6 hours and artificially aging them for 24 hours, the germination rates of the R31 and R80 seeds were enhanced. In conclusion, caffeic acid has been shown to partially mitigate the negative effects of soybean seed aging stress and to improve seed vitality. This finding should serve as a theoretical foundation for future research on the aging mechanism of soybean seeds.

## 1 Introduction

Soybean (*Glycine max* L. Merr.), the most important agricultural legume, was first planted in China around 5000 years ago (Sedivy et al., 2017). Today, it is grown all over the world and supplies 28% of vegetable oil and 70% of the protein meal consumed globally (SoyStats, 2021). Since soybean has such high nutritional and economic importance, conserving its genetic variety and gathering its germplasm resources have been the top goals for breeders (Lin et al., 2022). To address the rising need for plant proteins, oils, and food, however, we must breed soybean germplasm with improved performance due to climate change and population expansion.

An important component of sustainable agricultural production is the viability and longevity of high-quality seeds during storage, and seed longevity is the ability of seeds to germinate after they have been stored dry. The ripening and storage of seeds is a complicated process affected by several internal and external influences (Ramtekey et al., 2022). The seed is the primary means of plant reproduction and represents a crucial developmental stage with several unique characteristics. The preservation of plant biodiversity and the success of crops are both significantly hampered by seed life. Seeds have a variety of mechanisms (protection, detoxification, and repair) to survive in dry conditions and maintain a high germination capacity. As a result, the seed system offers a useful model for researching lifespan and aging (Rajjou and Debeaujon, 2008).

The vigor of crop seeds is crucial for maintaining the germplasm and enhancing grain quality. Wang et al. conducted a thorough analysis of the transcriptome and metabonomics of two subspecies of rice with varying levels of seed vigor obtained through sped-up senescence. They discovered that *bZIP23* is most likely to influence seed vigor through a common pathway with *PER1A* and that overexpressing and knocking out these two genes increased and decreased seed vigor, respectively (Wang et al., 2022b). For crop yield, resistance to seed aging and quick seedling development are crucial agronomic features. In comparison to the null segregant (NS) control, maize seedlings grew more quickly after germination due to the hyperaccumulation of IAA in the zygotic embryo of *zmdreb2a*. Additionally, the *zmdreb2a* seeds showed reduced seed aging tolerance due to reduced raffinose levels and decreased expression of *RAFFINOSE SYNTHASE (ZmRAFS)* in their embryos (Han et al., 2020). The longevity and vigor of seeds during seed maturation and germination in peas, soybeans, and *Medicago truncatula* are determined by *RFO* levels and the expression of genes that influence its synthesis, such as ABI5, raffinose synthase, and galactinol synthase (Salvi et al., 2016; Zinsmeister et al., 2016; Pereira Lima et al., 2017).

Numerous difficulties with manufacturing, post-harvest storage, and subsequent quality are always present in seeds. In addition, due to climate change, various stressors may result in subpar seed performance, such as decreased germination, uneven seedling emergence, subpar seedling establishment, and destructive changes in the root cell structure, significantly reducing yield (Reed et al., 2022).

Seeds may age more slowly in the wild than in artificial environments, which makes it difficult to understand the physiological mechanism. Thus, the normal aging process of mimicked seeds is accelerated by artificial conditions (Wang et al., 2016, 2022). It offers a scientific foundation for an in-depth examination of seed physiology and quality control procedures, and it aids in our understanding of the physiological changes that occur in seeds as they mature (Ku et al., 2014).

An increased storage time reduces seed vigor, and high-vigor seeds have better yield potential than low-vigor seeds. Utilizing artificial aging technology, we examined the physiological alterations and associated molecular processes of soybean seed aging by artificially aging two soybean lines with varying levels of vigor (‘R80’ and ‘R31’). We also combined transcriptome and metabolomics data analysis. This work offers fresh perspectives on safeguarding and utilizing germplasm resources and serves as a theoretical foundation for further research on the biology of soybean seeds.

## 2 Materials and methods

### 2.1 Plant materials and artificial aging conditions

The wild soybean ZYD00006 was used as the recipient parent, and the soybean cultivar SN14 as the recurrent parent. A population of 213 chromosome segment substitution lines (CSSL) was assembled using hybridization, backcrossing, and selfing. In 2020, the population was seeded in an experimental field in Gongzhuling City, Jilin Province, China. The following were used: a randomized block design, 15 cm plant spacing, 65 cm row spacing, and field management based on traditional soybean production in the area. To mimic the aging process, an artificial aging box was employed. A humidifier holding 4–5 L of distilled or filtered water was connected to a thermostat. From each variety, 600 seeds of a full and uniform size were randomly chosen, packaged in small nylon mesh bags, and placed on the net rack in an artificial seed aging box (LH-150S) (Xin et al., 2016; Zheng et al., 2022).

Before use, the aging box was cleaned with 75% ethanol. The appropriate quantity of sterilized or purified water was added to the water tank, and the age box was opened. The temperature and humidity for the aging period were set at 45°C and 95% relative humidity, respectively. The aging process occurred for 24, 72, and 120 hours. Finally, the seeds were air-dried naturally.

A germination test was conducted and significantly changed based on the germination conditions in the “International Seed Inspection Regulations.” A solution of 4% sodium hypochlorite was used to disinfect the seeds for 20–30 seconds before rinsing them 3 times in sterilized water. Forty soybean seeds from each variety were randomly chosen and placed in a glass Petri dish. Water was slowly added until a thin water film was visible on the paper. The seeds were covered with a layer of filter paper, and more water was added to moisten it (three instances). In an artificial incubator kept at a constant temperature of 25°C and in complete darkness, seeds were allowed to germinate. The number of germinated seeds was counted every day. The water absorbed by the seeds was replaced with sterile water. Germination was determined as follows: the radical be longer than half of the seed; if the radicle was spiral, the seed was not counted as germinated; and the number of germinated seeds was recorded each day for 7 days. Based on the results of standard germination tests and germination tests conducted after 96 hours of artificial accelerated aging (**Supplementary Table S1**), a total of 213 CSSL soybean populations were used as experimental materials. The stable phenotypic soybean lines R80 and R31, which had the highest anti-aging and aging sensitivity, respectively, were screened. Transcriptomic and metabolomic analyses of unaged R80 and R31 seeds and R31 and R80 seeds aged 24, 72, and 120 hours were performed. For each group, three biological repetitions were conducted for transcriptomic, and six biological repetitions were conducted for metabolomic.

### 2.2 RNA isolation and sequence analysis

Following the manufacturer’s instructions, total RNA was extracted using TRIzol reagent (Invitrogen, Carlsbad, CA, USA). Using Nanodrop, Qubit 2.0, and Agilent 2100 devices, the purity, concentration, and integrity of the RNA samples, respectively, were determined. The poly(A) technique was employed to enhance the mRNA once the samples were qualified. After the mRNA was reverse transcribed using oligo (dT) primers, the cDNA was broken apart. RNA-seq was performed by Biomarker Bioinformatics Technology (China) using a Hiseq 4000 PE150 sequencing technology to sequence and analyze the RNA samples (Illumina, San Diego, CA, USA). HISAT2 (Kim et al., 2015) (version 2.0.5) with default settings was used to map the raw sequencing reads to the soybean genome (v2) after being filtered using FASTP (Chen et al., 2018). DESeq2 (version 1.20.0) (Love et al., 2014) was used to identify differentially expressed genes (DEGs) (defined as those with an absolute value of expression fold change ≥ 2 and an FDR ≤ 0.05). **Supplementary Table S2** provides the primers used for qPCR to concurrently measure the transcript levels of genes associated with lipoxygenase (LOX).

### 2.3 Metabolite profiling

Biomarker Biotechnology Co., Ltd. (Beijing, China) conducted the non-targeted metabolome study. In summary, 300 μL of 75% methanol/water was added to 100 mg of material in a 1.5 mL centrifuge tube, and the mixture was centrifuged at 12,000 rpm for 10 minutes at 4°C. The Metlin database was used to identify every metabolite. An orthogonal partial least squares-discriminant analysis model was used to identify the differential metabolites. It had a variable importance of projection (VIP) score of ≥ 1 and a |log2 (fold change)| of ≥1. Utilizing the Kyoto Encyclopedia of Genes and Genomes (KEGG) database (http://www.kegg.jp/kegg/compound/), the functional annotations of these metabolites were acquired.

### 2.4 Enzyme, metabolite, and gene expression changes as seeds age

H_2_O_2_ content and peroxidase (POD) activity were first examined to evaluate aging-related cell damage or seed degeneration. Seeds (0.2 g) were used to determine the H_2_O_2_ concentration following the technique of Doulis et al (Doulis et al., 1997), and the results were computed as mol H_2_O_2_ decomposition min/1 g/1 FW. The thiobarbituric acid reaction technique, as reported by Gao et al. (Gao et al., 2008), was used to measure POD activity, which was determined using assay kits (Comin, Suzhou, China). LOX activities contributed to seed healing mechanisms. To obtain enzyme crude extract with 0.1 mM potassium phosphate buffer, three replications of the same procedure were performed using approximately 0.1 g of seed each time (pH 7.8). When performing enzyme activity tests, the supernatant was kept at 4°C. As previously mentioned, sodium phosphate reaction buffer (150 mM, pH 8.0) and linoleic acid substrate solution (10 mM linoleic acid) were produced for the LOX tests (Stephany et al., 2015). A UV spectrophotometer was used to measure the LOX reaction of 0.1 g of seeds at 234 nm.

## 3 Results

### 3.1 Dissecting the transcriptome profiles of seed with different levels of vigor

This study’s artificial accelerated aging induction experiment was used to assess the seed vigor. According to the characteristics of seed germination on aging stress, R31 lost its seed germination ability, while R80 maintained a cumulative germination rate of 64.44% after 96 hours of treatment (**Table 1**). Based on the findings, R31 was an aging-sensitive line, and R80 was an aging-resistant line.

**Table 1:** Modification of seed germination in R31 and R80 after accelerated aging treatment.

Treatment	ID	Repetition1	Repetition2	Repetition3	Average germination rate
CK	R31	90.00%	93.33%	100.00%	94.44%
CK	R80	93.33%	96.67%	70.00%	86.67%
Aging	R31	0.00%	0.00%	0.00%	0.00%
Aging	R80	60.00%	63.33%	70.00%	64.44%

The seeds were aged at 95% RH and below 45°C.

**Figure 1:**
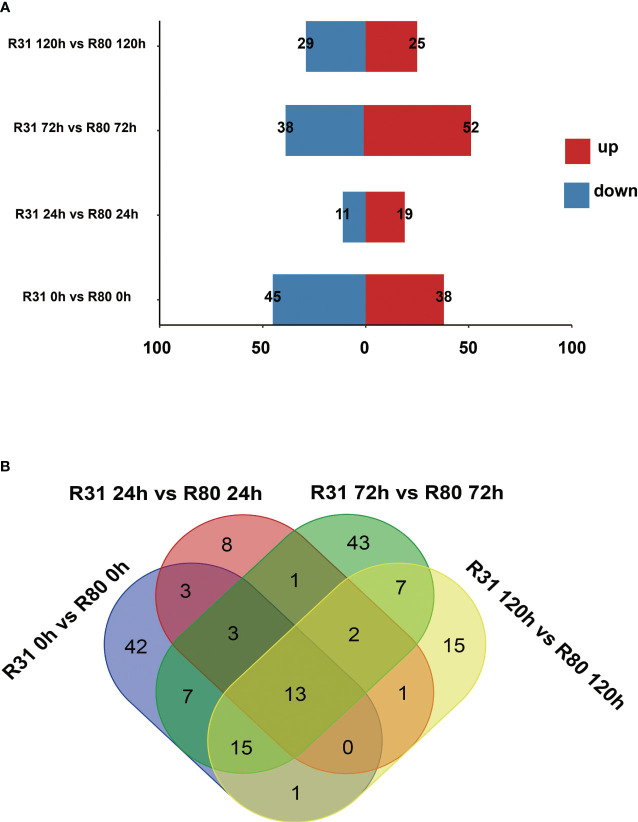
Statistics on the number of differentially expressed genes (DEGs) in seeds under aging stress **(A)**; Venn diagram of changes in differentially expressed genes (DEGs) in R31 and R80 seeds under aging stress **(B)**.

The variation in seed vigor between R31 and R80 may be influenced by changes in gene expression. The correlation study revealed that at various stages of artificial aging stress, all biological repetitions of the group internal sample of the R80 or R31 lines showed relatively consistent gene expression levels (**Supplementary Table S3**). Our study examined the expression profiles of two soybean lines (R31 and R80) that differed in their ability to withstand the effects of aging. Four comparison groups were created: R31 0 h versus R80 0 h, R31 24 h versus R80 24 h, R31 72 h versus R80 72 h, and R31 120 h versus R80 120 h. In the four comparison groups, 83 (38 upregulated and 45 downregulated), 30 (19 upregulated and 11 downregulated), 90 (52 upregulated and 38 downregulated), and 54 (25 upregulated and 29 downregulated) differentially expressed genes (DEGs) were identified respectively (**Figure 1A**; **Supplementary Table S4**).

**Figure 2:**
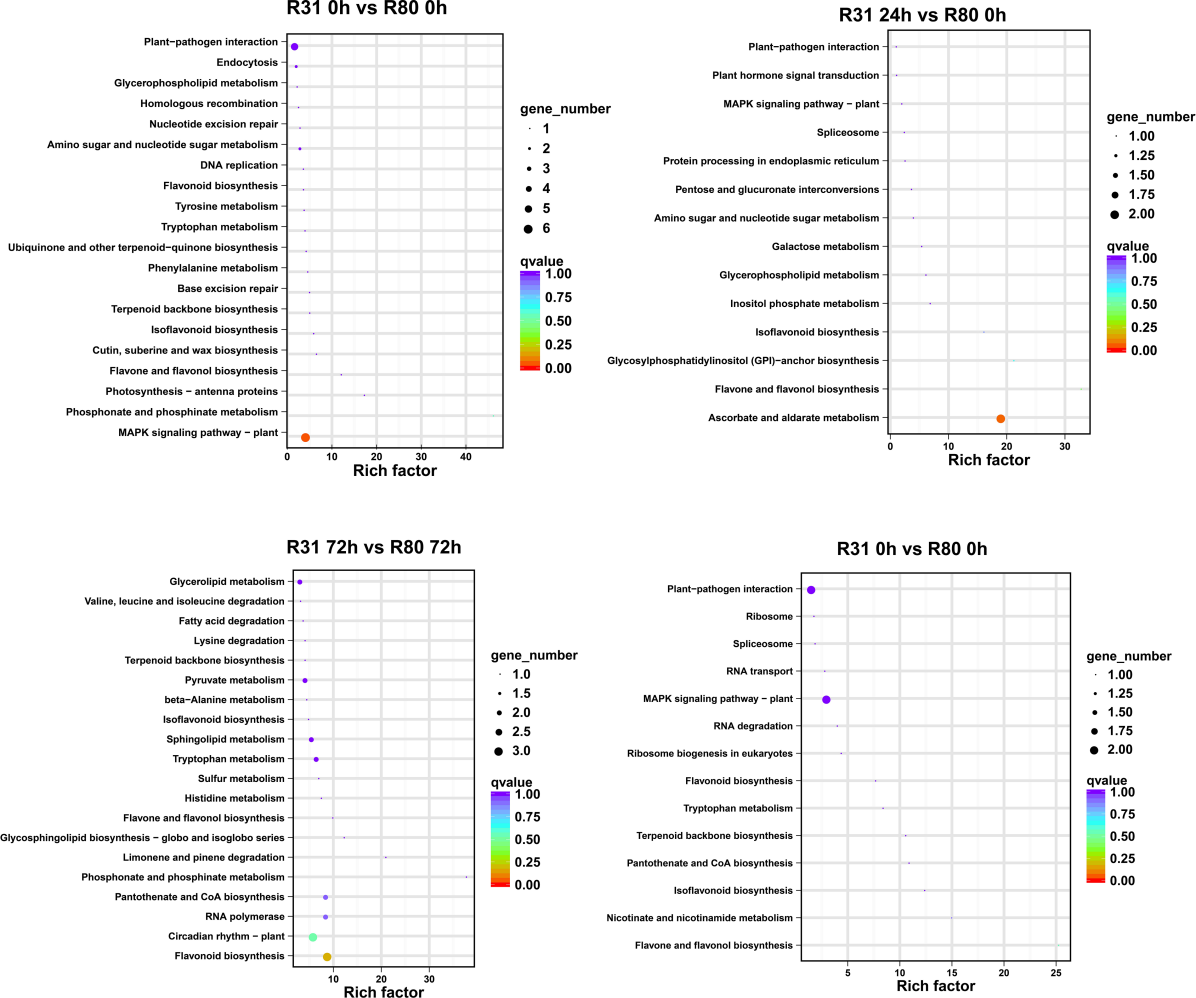
KEGG pathway changes in aged seeds of R31 and R80.

The four artificial aging therapy groups shared 13 DEGs (**Figure 1B**). In addition, 12 of the 13 DEGs consistently exhibited upregulation and just one consistently showed downregulation in each comparison group (**Supplementary Figure S1**; **Supplementary Table S4**). KEGG functional enrichment analysis was then performed on the DEGs between R80 and R31. The DEGs between the non-aged groups (R80-0 h versus R31-0 h) were mainly enriched in pathways including flavonoid biosynthesis, circadian rhythm—plant, RNA polymerase, pantothenate and CoA biosynthesis, phosphonate and phosphinate metabolism, limonene and pinene degradation, glycosphingolipid biosynthesis—globo and isoglobo series, flavone and flavonol biosynthesis, histidine metabolism, sulfur metabolism, tryptophan metabolism, sphingolipid metabolism, isoflavonoid biosynthesis, beta-alanine metabolism, pyruvate metabolism, terpenoid backbone biosynthesis, lysine degradation, fatty acid degradation, valine, leucine and isoleucine degradation, and glycerolipid metabolism (**Figure 2**). DEGs between aged seed groups [(R31 0 h versus R80 0 h, R31 24 h versus R80 24 h, R31 72 h versus R80 72 h, and R31 120 h versus R80 120 h) were mostly related to phosphonate and phosphinate metabolism, MAPK signaling pathway—plant, glycerolipid metabolism, valine, leucine and isoleucine degradation, fatty acid degradation, lysine degradation, pyruvate metabolism, beta-alanine metabolism, sphingolipid metabolism, sulfur metabolism, histidine metabolism, glycosphingolipid biosynthesis—globo and isoglobo series, limonene and pinene degradation, pantothenate and CoA biosynthesis, RNA polymerase, circadian rhythm—plant, plant hormone signal transduction, spliceosome, protein processing in endoplasmic reticulum, pentose and glucuronate interconversions, galactose metabolism, inositol phosphate metabolism, glycosylphosphatidylinositol—anchor biosynthesis, and ascorbate and aldarate metabolism (**Figure 2**). Remarkably, the KEGG enrichment study (**Figure 2**), before and after artificial aging therapy, showed that R31 and R80 had different metabolic pathways, especially with regard to flavonoid, flavonoid, and flavanol metabolism. Between the two lines, these metabolic pathways continuously varied (**Table 2**; **Figure 2**; **Supplementary Figure S2**). The transcriptome study also revealed that aging treatment caused a greater transcriptional difference between R80 and R31 seeds, explaining the increase in anti-aging processes in R80 seeds.

**Table 2:** List of DEGs involved in phenylpropanoid metabolism pathway during aging of R31 and R80 seeds.

Gene_name	R31 0 h versus R80 0 h	R31 24 h versus R80 24 h	R31 72 h versus R80 72 h	R31 72 h versus R80 72 h	KEGG_pathway_annotation
Glyma.19G254600	Down	Normal	Down	Down	Flavonoid biosynthesis
Glyma.08G247100	Up	Up	Up	Up	Isoflavonoid biosynthesis; flavone and flavonol biosynthesis
Glyma.01G228700	Normal	Normal	Up	Normal	Flavonoid biosynthesis; circadian rhythm—plant
Glyma.11G011500	Normal	Normal	Up	Normal	Flavonoid biosynthesis; circadian rhythm—plant

### 3.2 KEGG analysis of differentially accumulated metabolites in seed during aging stress

**Figure 3:**
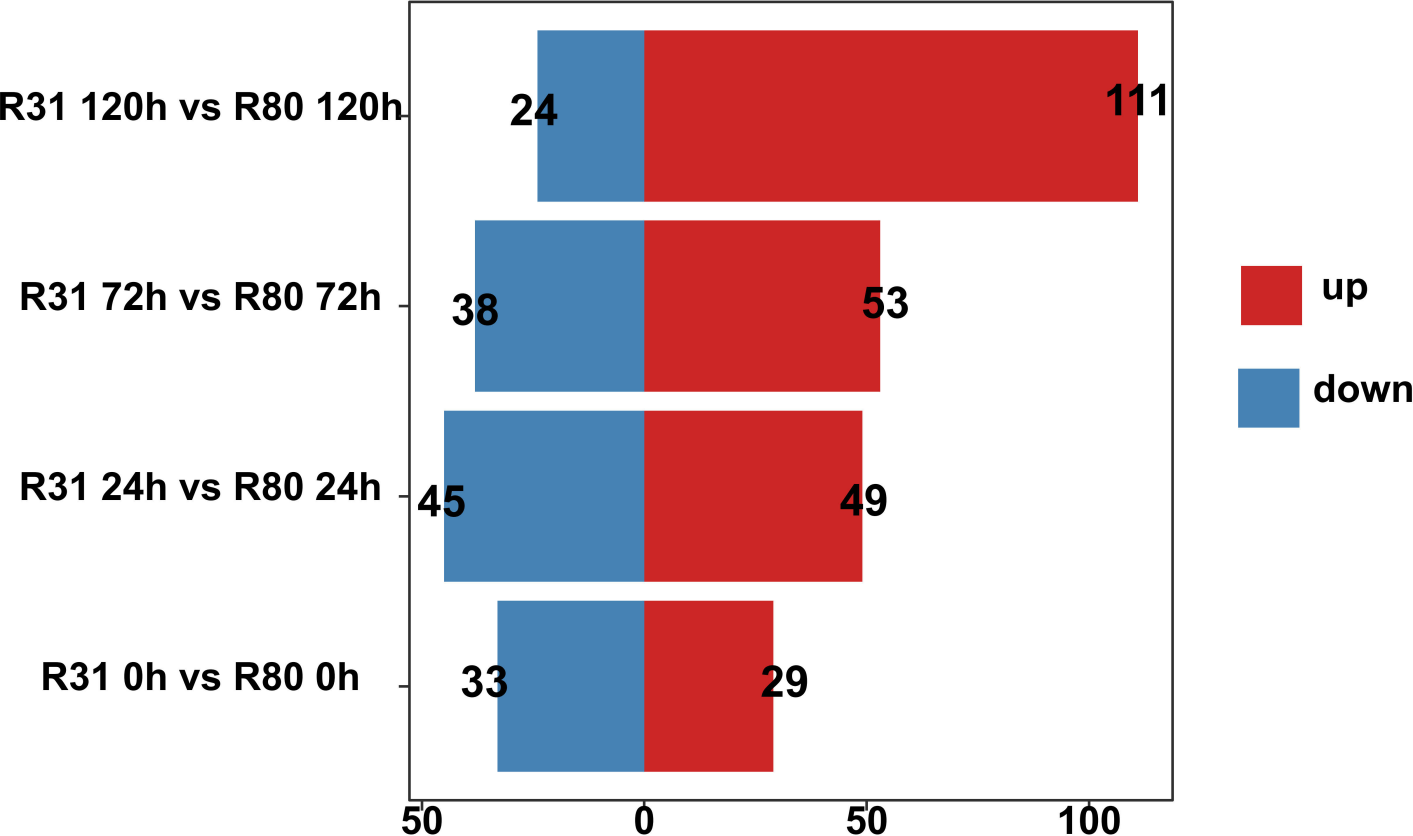
Statistics on the quantity of differentially accumulated metabolites for seeds under aging stress.

The purpose of artificially accelerating aging was to identify the metabolite differences between the R31 and R80 seeds. The R31 and R80 seeds used in this study were matured for 0, 24, 72, and 120 hours before being used as samples for Qualcomm quantitative metabolite analysis. After metabolomics data analysis, 799 metabolites were discovered. The correlation study revealed that at different stages of artificial aging stress, all biological repetitions of the group internal sample of the R80 or R31 lines showed relatively consistent accumulated metabolite patterns (**Supplementary Table S5**). Four comparison groups were created and measured in total. The control group was the non-aged group, and the treatment groups comprised three artificially aged groups, with aging for 24, 72, and 120 hours. There were 62 (29 upregulated and 33 downregulated), 94 (49 upregulated and 45 downregulated), 91 (53 upregulated and 38 downregulated), and 135 (111 upregulated and 24 downregulated) differentially accumulated metabolites (DAMs) in the four comparison groups, respectively, identified using a VIP score of 1 and an absolute multiple change of 2 (**Figure 3**).

**Figure 4:**
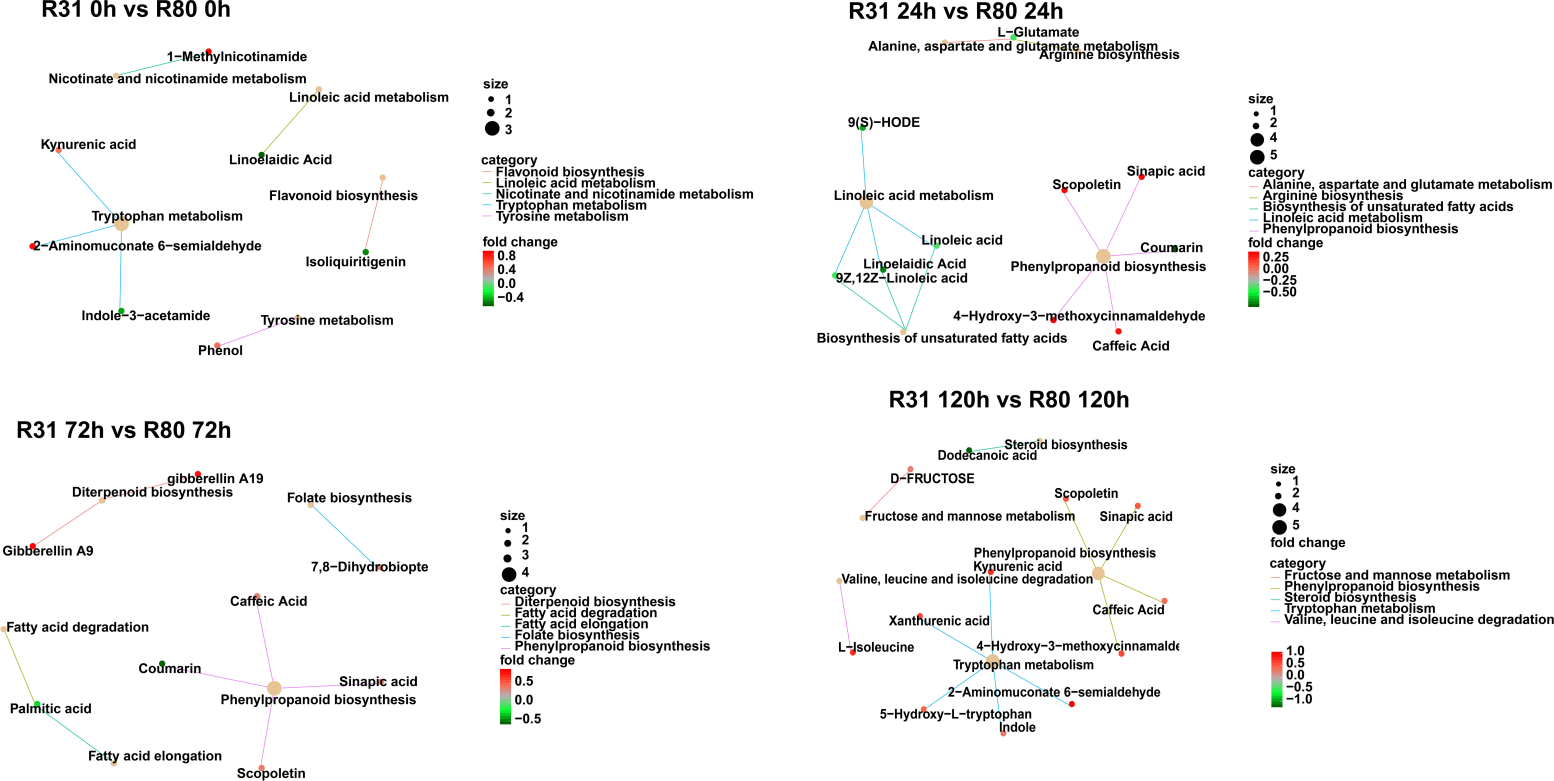
KEGG analysis of differentially accumulated metabolites (DAMs) in R31 and R80 seeds under aging stress.

**Figure 5:**
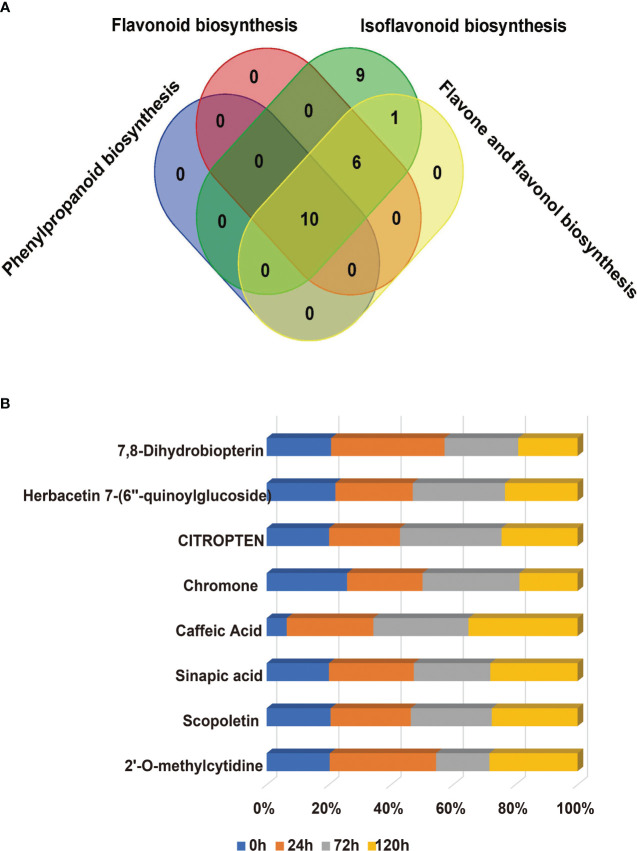
Venn diagram of the changes of differentially accumulated metabolites (DAMs) related to phenylpropanoid metabolism pathway in R31 and R80 seeds under aging stress **(A)**; Compared with R31, the increasing proportion of different accumulated metabolites in R80 changed with aging time **(B)**.

The KEGG enrichment indicates that the aging process of the R31 and R80 seeds drastically changed several metabolic pathways. These pathways included phenylpropanoid biosynthesis, flavonoid biosynthesis, tryptophan metabolism, diterpenoid biosynthesis, fatty acid degradation, fatty acid elongation, linoleic acid metabolism, biosynthesis of unsaturated fatty acids, alanine, aspartate, and glutamate metabolism (**Figure 4**). With the use of metabolic analysis network technologies, a metabolic network was created to investigate any possible relationships between these metabolites. These metabolites are associated with the key nodes of the network involved in phenylpropanoid biosynthesis, flavonoid biosynthesis, isoflavonoid biosynthesis, flavone biosynthesis, and flavonol biosynthesis (**Figure 5A**). Several cumulative metabolites (DAMs) were identified, including caffeic acid, coumarin, sinapic acid, scopoletin, 4-hydroxy-3-methoxycinnamaldehyde, and isoliquiritigenin (**Figures 5A**; **Supplementary Table S6**). Unexpectedly, during aging stress, phenylpropanoid and flavonoid levels rose, particularly during dynamic variations in the concentration of caffeic acid. The results indicated that the caffeic acid concentration of the R80 seeds exhibited an increasing tendency and remained greater than that of the R31 seeds with the extension of artificial aging time (**Figure 5B**). The experimental data mentioned earlier suggest that caffeic acid may be a significant factor in the functional modulation of seed vigor under aging stress.

### 3.3 Activities of LOX and antioxidant enzymes

**Figure 6:**
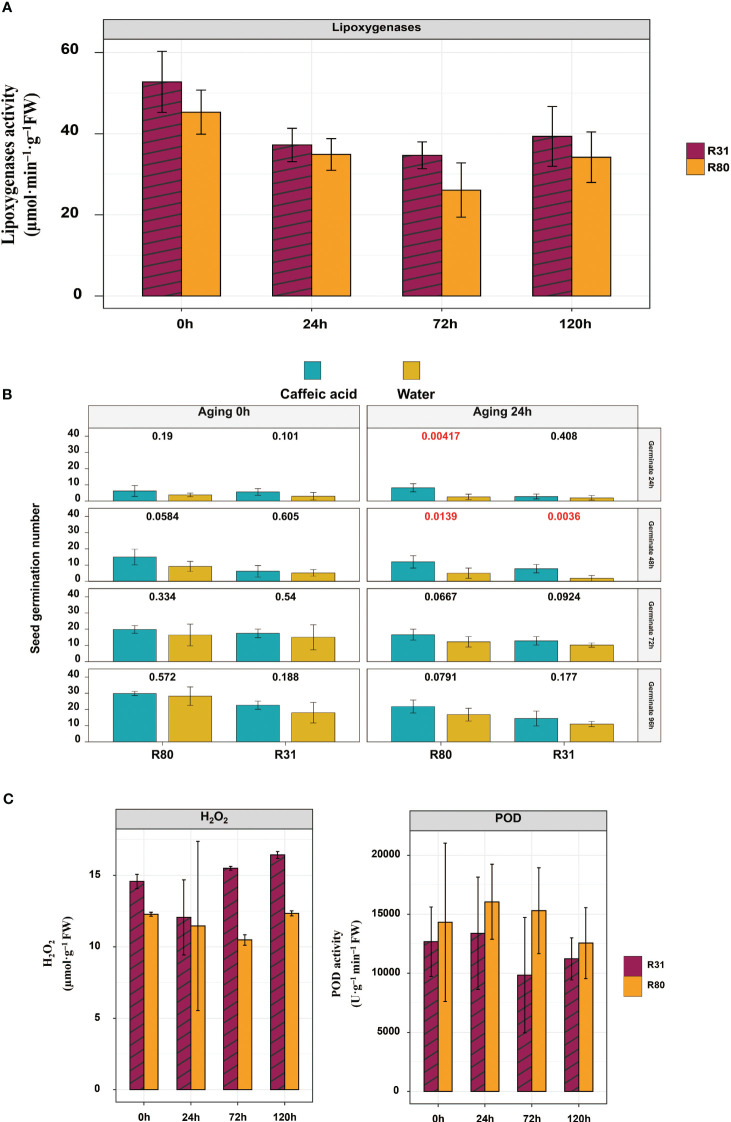
Lipoxygenase (LOX) activity of R31 and R80 seeds during aging stress **(A)**; inhibition of LOX activity increased the vigor of R31 and R80 seeds under artificial aging stress (seed number n = 30) **(B)**; and seed aging increased the contents of endogenous H_2_O_2_ and POD in R31 and R80 seeds **(C)**.

**Figure 7:**
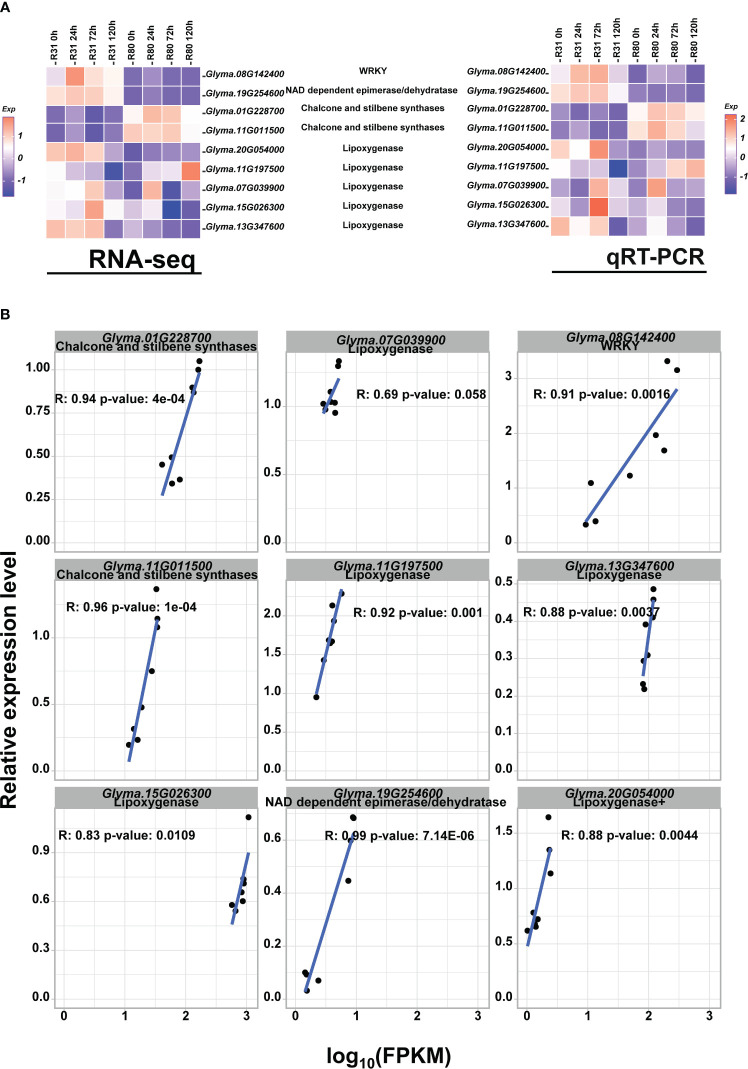
qRT-PCR verified the accuracy of RNA-seq in detecting gene expression levels. Heatmap of RNA-seq and RT-PCR gene expression levels for the randomly selected DEGs. **(A)**; Correlation analysis of RNA-seq and RT-PCR gene expression levels for the randomly selected DEGs **(B)**.

Reactive oxygen species (ROS) accumulation, ascorbic acid-glutathione circulatory activity decline, and mitochondrial function delay all contribute to mitochondrial dysfunction in aged soybean seeds (Xin et al., 2014). Excess ROS buildup in mitochondria results in the breakdown of the antioxidant system, which is the cause of seed degeneration (Kurek et al., 2019). Two soybean cultivars, ‘R80’ and ‘R31,’ were shown to have endogenous H_2_O_2_ levels that increased with age. Notably, R31 seeds did not exhibit aging resistance, but had much greater endogenous H2O2 concentrations than R80 seeds. Compared to the sensitive aging variety R31, the endogenous H2O2 level of age-resistant variety R80 decreased by 5.28, 47.78, and 33.09% after rapid artificial aging for 24, 72, and 120 hours, respectively. In contrast, the endogenous POD level of the aging-tolerant variety R80 increased by 55.56, 19.92, and 12.93% after rapid artificial aging for 24, 72, and 120 hours, respectively, compared to the sensitive aging variety R31. Enzymes play crucial roles in the growth and development of plant life. The LOX family of enzymes is one of the most important. During seed storage, LOX can catalyze the oxidation of unsaturated fatty acids and produce hydroperoxide, which reduces the vigor and nutritional quality of seeds (Viswanath et al., 2020). Seed vigor, antioxidant enzymes, and LOX activities, along with corresponding gene expression, were evaluated after aging to gather more insight into the possible mechanisms behind seed deterioration and to validate the idea that LOX activity might be a new sensitive signal for predicting seed aging during storage. According to the findings, under artificially generated aging stress, the R80 seeds showed reduced LOX activity compared with the R31 seeds (**Figure 6A**). Moreover, the R80 seeds had lower levels of LOX gene expression than the R31 seeds (**Figure 7A**). LOX activity suppression research is required to comprehend how soybean seeds age and to find a way to postpone seed aging and extend seed life. Furthermore, LOX activity was considerably inhibited by a few phenolic substances, with caffeic acid being the most potent inhibitor (approximately 57% of inhibition) (Szymanowska et al., 2009). Additionally, by soaking R31 and R80 in 1 mM caffeic acid for 6 hours and artificially aging them for 24 hours, the germination rates of these seeds were raised. These results showed that LOX promoted the aging of soybean seeds and that reducing LOX activity preserved the vitality and viability of aging seeds.

### 3.4 qRT-PCR verified the accuracy of RNA-seq in detecting the gene expression level

In this work, the accuracy of transcriptome data in determining gene expression levels was confirmed using qRT-PCR. Four genes encoding DEGs and five genes encoding LOX were chosen at random. The findings demonstrated that the transcriptome data of these chosen genes were highly consistent with the qRT-PCR results (**Figure 7A**) and that the qRT-PCR results and the relative expression level of log _10_(FPKM) of nine genes obtained by RNA-seq exhibited a nearly linear correlation (**Figure 7B**).

## 4 Discussion

Seeds are essential for crop growth, human nutrition, and food security. The primary elements influencing crop seed function are the intricate features of seed vigor. Successful planting is critical to crop productivity and resource efficiency. The strength, regularity, and speed at which seeds germinate and generate seedlings under a variety of environmental conditions are determined by their vigor (Finch-Savage and Bassel, 2015). A chemical fingerprint, or signature of an organism’s or sample’s metabolic condition at a particular moment in time, can be obtained through metabolomic analysis. The metabolites to be examined may be the end or intermediate products of plant metabolic pathways, or they may arise due to environmental influences from the outside world (Shen et al., 2022). Extreme flexibility in phenylpropanoid metabolism can result in a large variety of products that function in plant growth and interactions with the environment in response to various developmental stages and constantly shifting environmental variables (Yuan and Grotewold, 2020). Seed permeability and resistance to mechanical damage are correlated with the lignin concentration of the soybean seed coat (Capeleti et al., 2005).

The seed coat is a type of composite structure that may be used as a conduit for nourishment obtained from growing embryos. The seed coat offers the embryos shelter and protection once the seeds have dried and matured. It can also apply dormancy or cause germination by regulating water absorption. The qualities of the seeds as a whole and the usefulness of their derivatives will be influenced by the features established by the seed coat for crops such as soybean. Recently, fascinating instances of atypical genetic pathways regulating seed coat breaking, gloss, and color have been identified in soybean (Qutob et al., 2008). Plant physiology depends on a large class of secondary plant metabolites generated from phenylalanine. Seed coats contain phenolic chemicals that make the seed harder and prevent microbial development. The seed coat shields the seed from electrolyte leaks and hydration stress during germination (Mohamed-Yasseen et al., 1994). In the current investigation, we found that the seed vigor of line R80 was superior to that of line R31 under the stress of aging (**Table 1**). Natural phenolic compounds include phenylpropanoids. They are a broad group of phenylalanine-derived secondary plant metabolites that are essential to plant physiology (Dong and Lin, 2021). These substances serve as crucial cell wall building blocks, shielding plants from a variety of biotic and abiotic environmental stressors (Treutter, 2006; Vogt, 2010; Kiani et al., 2021). Integrating the data from transcriptome and metabolome analyses showed that the DAMs and DEGs of the four comparison groups (R80-0 h versus R31-0 h, R31-24 h versus R80-24 h, R31-72 h versus R80 72 h, and R31-120 h versus R80-120 h) were mapped to the KEGG database. Phenylpropanoid, flavonoids, isoflavones, flavonoids, and flavonols were among the co-mapped pathways. Particularly those connected to the metabolite of seed coat known as caffeic acid were responsible for the difference between R31 and R80 response to aging stress (**Figures 2**, **4**).

Seeds have evolved extraordinarily effective repair systems, including enzymatic antioxidant systems, to achieve homeostasis of H_2_O_2_ production (Xia et al., 2015). During lipid peroxidation in the seeds of rice (*Oryza sativa* L.) and soybean (*Glycine max* (L.) Merr.), LOX (LOX, EC1.13.11.12) plays a significant role (Lima et al., 2010; Xu et al., 2015). When artificially generated aging stress was applied to the R80 seeds, LOX activity was lower than that in the R31 seeds (**Figure 6A**). Both internal metabolic processes and external stimuli cause the generation of ROS, including superoxide anion radicals, hydrogen peroxide, and hydroxyl radicals, in all cells. Nonetheless, by activating several antioxidant mechanisms, cells are often able to lower the oxidative potential of ROS. Several substances, including caffeic acid and its derivatives, have been shown to possess antioxidant qualities (Nardini et al., 2001). In artificially aged soybean seeds, LOX activity was inhibited by the application of caffeic acid. Additionally, the viability of the R80 and R31 seeds improved dramatically after 24 hours of artificial aging, suggesting that caffeic acid may strengthen the seeds’ resistance to storage (**Figure 6B**). In contrast to the sensitive aging variety R31, the aging-tolerant variety R80 demonstrated that after rapid artificial aging for 24, 72, and 120 hours, the endogenous levels of H_2_O_2_ fell by 5.28, 47.78, and 33.09%, respectively (**Figure 6C**).

In plant research, there has been a great deal of interest in the detection and characterization of differential gene expression from tissues exposed to stress. The likelihood of advancing crop improvement by direct genetic modification increases with the identification of components involved in the response to a certain stress (Dunwell et al., 2001). Previous work discovered a novel WRKY transcription factor, *OsWRKY29*, that adversely controls rice seed dormancy. *OsWRKY29* overexpression decreased seed dormancy, whereas its knockout and RNA interference increased it (Zhou et al., 2020). For instance, transcription factors such as WRKY3 and NFLX1, which are involved in plant defense, also have an influence on seed survival by controlling the permeability of the seed coat (Debeaujon et al., 2000). This study’s findings demonstrated that under normal conditions, the expression level of the WRKY (*Glyma.08G142400*) transcription factor in R80 was much lower than that of R31. Thus, we concluded that R80 exhibited low WRKY expression (**Figure 7A**), which helped R80 seeds become dormant and avoid the negative effects of aging stress.

As a consequence of inter-cultivar vigor fluctuations and artificial aging, we discovered numerous potential metabolites and related DEGs by the interactive comparison of transcriptomic and metabolomic data. The genetic and metabolic underpinnings of inter-cultivar vigor variations and artificial soybean seed aging are better understood because of this research.

## 5 Conclusion

Maintaining genetic variety and germplasm resources has become a major concern for breeders to achieve seed quality and germplasm preservation. This study compared and examined the transcription and metabolic data of two soybean lines treated with aging stress: R31 (aging sensitive) and R80 (aging tolerant). Four sets of artificial aging treatments—0, 24, 72, and 120 hours—with varying durations were performed. There were differences in the DEGs and differentially accumulated metabolites between the two soybean lines aged for different durations. The ability of soybean seeds to withstand the effects of age has been revealed to be mostly regulated by the phenylpropanoid metabolic pathway, particularly caffeic acid. Longer aging treatment periods produced higher levels of caffeic acid, and this buildup enhanced the seed’s anti-aging ability by blocking LOX activity. Moreover, the germination rates of the R31 and R80 seeds were increased by immersing them in 1 mM caffeic acid for 6 hours and artificially aging them for 24 hours. Overall, this study indicates that the detrimental effects of seed aging stress may be somewhat mitigated and that seed vitality can be improved by the accumulation of metabolites, specifically caffeic acid, in the soybean phenylpropanoid acid metabolic pathway. The results of this study offer a theoretical framework for further investigations into the mechanism of soybean seed aging.

## Data availability statement

The datasets presented in this study can be found in online repositories. The names of the repository/repositories and accession number(s) can be found below: https://www.ncbi.nlm.nih.gov/, PRJNA1112530.

## Author contributions

GL: Writing – original draft, Visualization. JX: Writing – original draft, Data curation. WZ: Funding acquisition, Resources, Writing – review & editing. FM: Writing – review & editing, Investigation. MY: Validation, Writing – review & editing. X-HF: Formal analysis, Writing – review & editing. XS: Investigation, Writing – review & editing. YHZ: Validation, Writing – review & editing. YFZ: Resources, Writing – review & editing. MW: Resources, Writing – review & editing. QC: Funding acquisition, Writing – review & editing. SW: Funding acquisition, Writing – review & editing. HJ: Funding acquisition, Writing – review & editing.

## Supplementary material

Supplementary Table 1 Germination rate of 213 CSSL soybean populations during a standard germination test and after a 96-hour artificial accelerated aging test.

Supplementary Table 2 Gene primer sequences used in qRT-PCR.

Supplementary Table 3 Correlation matrix analyses of gene expressions among biological replicates of different samples from different artificial aging conditions between the R80 and the R31 lines.

Supplementary Table 4 Differentially expressed genes of the four comparison groups.

Supplementary Table 5 Correlation matrix analyses of accumulated metabolites among biological replicates of different samples from different artificial aging conditions between the R80 and the R31 lines.

Supplementary Table 6 Differentially accumulated metabolites of the four comparison groups.
