# Supplementary material for: Integrated examination of the transcriptome and metabolome of the gene expression response and metabolite accumulation in soybean seeds for seed storability under aging stress
Source: Front Plant Sci. 2024 Jul 8;15:1437107. doi: 10.3389/fpls.2024.1437107 (PMC11261460; doi:10.3389/fpls.2024.1437107)
Supplement: Supplementary file 1 [file DataSheet_1.docx]

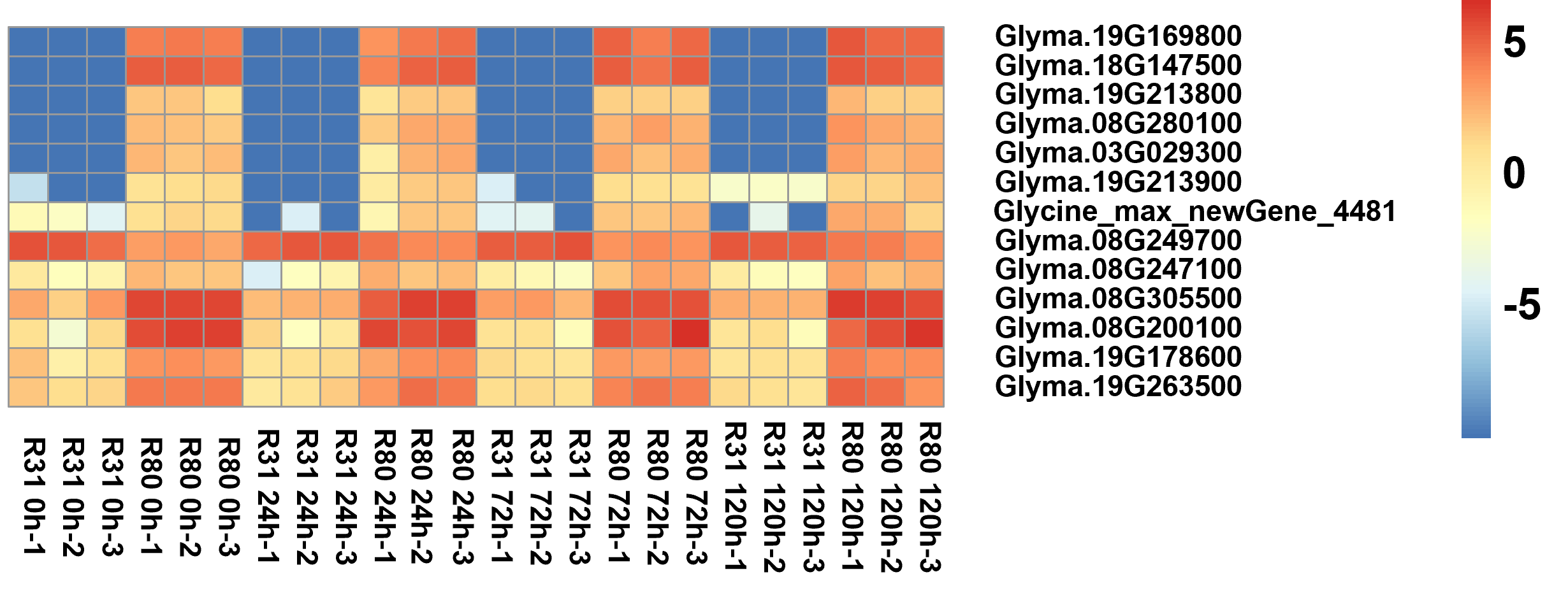


**Figure S1 Expression profile of 13 differentially expressed genes (DEGs) shared between R31 and R80 after seed aging.**


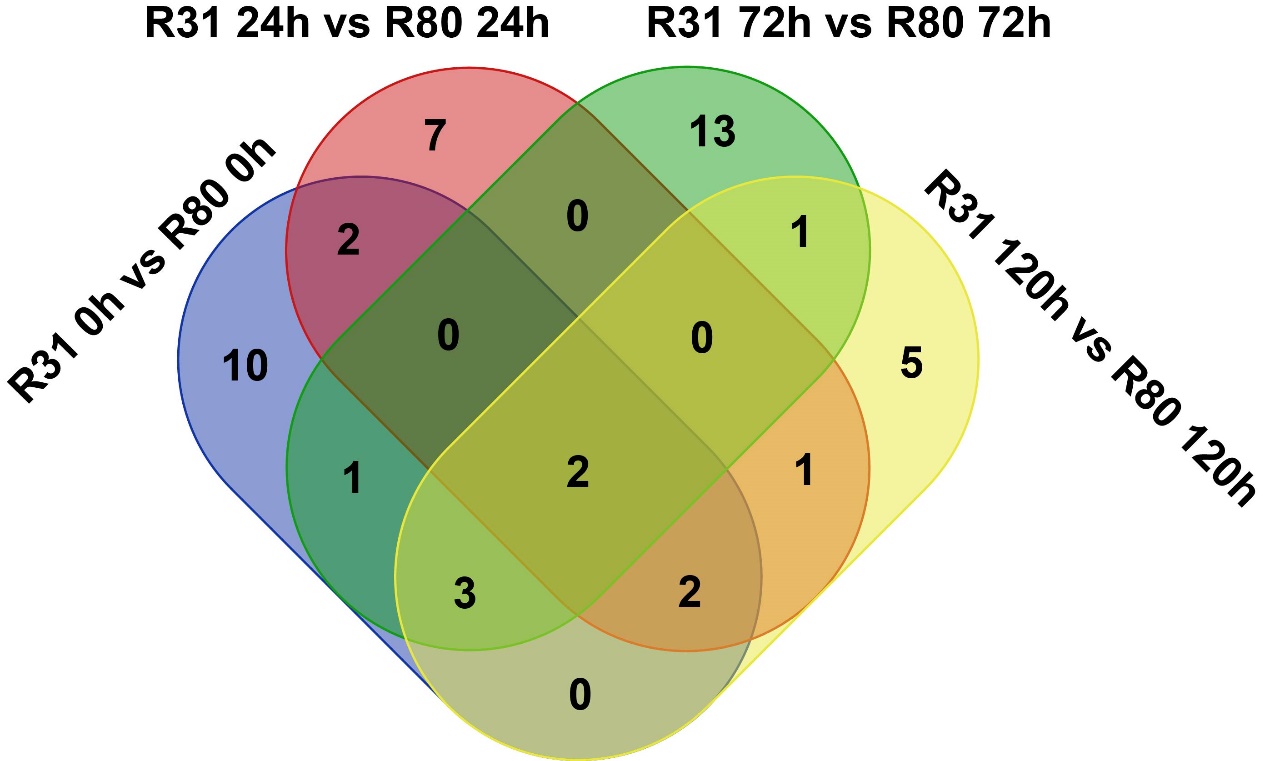


**Figure S2 Venn diagram showing how the KEGG pathway changed as R31 and R80 seeds aged.**
